# Increased Overall Survival and Decreased Cancer-Specific Mortality in Patients with Hepatocellular Carcinoma Treated by Transarterial Chemoembolization and Human Adenovirus Type-5 Combination Therapy: a Competing Risk Analysis

**DOI:** 10.1007/s11605-018-3703-3

**Published:** 2018-02-12

**Authors:** Chaobin He, Yu Zhang, Xiaojun Lin

**Affiliations:** 1 Department of Hepatobiliary and Pancreatic Surgery, State Key Laboratory of Oncology in South China, Collaborative Innovation Center for Cancer Medicine, Sun Yat-sen University Cancer Center, Guangzhou, Guangdong 510060 People’s Republic of China; 2 State Key Laboratory of Ophthalmology, Zhongshan Ophthalmic Center, Sun Yet-sen University, Guangzhou, Guangdong 510060 People’s Republic of China

**Keywords:** Hepatocellular carcinoma, Transarterial chemoembolization, Human type-5 adenovirus, Competing risk analysis, Cancer-specific mortality

## Abstract

**Background:**

In analyzing cancer patient survival data, the problem of competing risks is often ignored. This study used a competing risk approach to evaluate the efficacy of recombinant human type-5 adenovirus (H101) in patients with hepatocellular carcinoma (HCC) treated by transarterial chemoembolization (TACE).

**Methods:**

In this retrospective study, 476 patients were included. The cumulative probabilities of cancer-specific mortalities were analyzed by the Kaplan-Meier (KM) method and a competing risk model. Competing risk regression was used to assess the predictive factors for cumulative cancer-specific mortalities.

**Results:**

Two hundred thirty-eight HCC patients received combination TACE and H101 therapy, and another 238 HCC patients received TACE therapy alone. For patients in the TACE with H101 group, estimated 1-, 2-, and 3-year overall survival (OS) rates were 61.0, 40.0, and 31.5%, respectively, while for patients in the TACE group, the estimated 1-, 2-, and 3-year OS rates were 55.0, 33.4, and 22.3%, respectively. The 1-, 2-, and 3-year cancer-specific mortality rates for patients in the TACE with H101 group vs. the TACE group were 37.3 vs. 42.0%, 55.7 vs. 63.5%, and 61.9 vs. 74.7%, respectively. Multivariate competing risk analysis established that a combination of TACE and H101 therapy was an independent factor in decreasing cancer-specific mortality.

**Conclusions:**

Compared with TACE therapy, patients who were diagnosed with unresectable HCC treated with combined TACE and H101 therapy had increased OS and decreased cancer-specific mortality. The survival benefit was more obvious in patients with elevated AFP, absence of metastasis, single tumor, enlarged tumor, and HBsAg-positivity.

## Introduction

Hepatocellular carcinoma (HCC) is the fifth most common cancer and the third leading cause of cancer-related mortality worldwide.^1^ Several treatment procedures, including hepatectomy, liver transplantation, and radio-frequency ablation, are recommended for early-stage HCC.^2^,^3^ However, due to the lack of symptoms during the early stage,^4^ most of HCC cases are diagnosed at an advanced stage and unsuitable for curative therapy.^5^ Transarterial chemoembolization (TACE), which focuses on delivering chemotherapeutic drugs to the tumor while blocking tumor-feeding arteries, has shown a survival benefit for unresectable HCC.^6^,^7^ However, diminished liver function, tumor enlargement, and portal vein involvement may lead to reduced efficacy of TACE.^8^ Moreover, repeated TACE may also create a hypoxic microenvironment, which promotes tumor progression.^9^,^10^ The poor prognosis^11^ of unresectable HCC treated by TACE suggests that more improvements are needed to better benefit patients.

Genetic abnormalities are commonly observed during HCC formation, such as activated oncogenes^12^ and inactivated tumor suppressor genes.^13^ Deletion or mutation of wild-type p53 frequently occurs in HCC, indicating a poorer patient prognosis.^14^ H101, which is generated by both E1B and E3 gene deletions, is a recombinant human type-5 adenovirus.^15^ H101 infects tumor cells, ultimately killing them through viral oncolysis.^16^ The active p53 gene in normal cells prevents the adenovirus from replicating and lysing cells, leading to selective H101 replication in cancer cells, rather than normal cells. In addition, this selectivity leads to tumor cell cytolysis without adverse side effects.^17^ Tumor cell sensitivity to H101 in vitro is negatively reflected by the p53 gene sequence, which is thought to be due to p53 inactivation by several mechanisms.^18^ Furthermore, H101 enhances the cell-mediated immune and host immune systems, improving the efficacy of TACE treatment.^19^ In addition, adenovirus safety was improved by deleting a 78.3–85.8-nm gene segment in the E3 region that encodes the adenovirus death protein.^20^ Combined TACE and H101 therapy will likely benefit HCC patient survival^21^; however, no phase III clinical trials have shown a survival benefit for this combination therapy to date. Therefore, it is necessary to explore the value of combining H101 with TACE treatment in HCC patients.

Competing risk refers to an event that precludes another event under investigation or fundamentally alters the probability of the outcome of interest.^22^,^23^ In the survival analysis of HCC patients, a patient may experience cancer-specific death, non-cancer-specific death, survival, or lost to follow-up. Non-cancer-specific death is a competing risk event that prevents the event of interest, cancer-specific death. Failure to recognize the presence of a competing risk may result in misleading conclusions in clinical practice. In this case, it is unsuitable to use the Kaplan-Meier (KM) method to analyze survival data because this method treats competing events independently and overestimates the proportion of cancer-specific mortality. The cumulative incidence function (CIF)^24^ accounts for the informative nature of this censorship and corresponds to the probability of a particular event occurring without assuming of independence between event types and can be used to analyze survival data.

Competing risk analysis has been adopted to analyze several cancers, including nasopharyngeal,^25^ ovarian,^26^ kidney,^27^ and breast cancers.^28^ However, to our knowledge, no relative reports focus on this analysis in HCC patients. In the current work, a competing risk analysis was conducted to explore the therapeutic effects of combined TACE and H101 therapy in HCC patients.

## Patients and Methods

### Patients

Clinical data were collected using a cohort of consecutive patients who received TACE therapy as their initial treatment at the Department of Hepatobiliary and Pancreatic Surgery of Sun Yat-sen University Cancer Center between January 2007 and July 2015. HCC was diagnosed based on the typical features of HCC identified by two radiological images or one radiological image combined with elevated alpha-fetoprotein (AFP) levels (≥ 400 ng/mL) or histopathological evidence, which is consistent with the diagnostic criteria for HCC used by American Association for the Study of the Liver guidelines.^29^ The inclusion criteria for this study were as follows: (1) no previous treatment before TACE, (2) liver function Child-Pugh A or B, and (3) a follow-up period ≥ 1 year. The exclusion criteria were as follows: (1) liver function Child-Pugh C, (2) common diagnosis of secondary cancers, (3) any therapies other than TACE after initial TACE treatment, or (4) lost to follow-up.

### Data Collection

We reviewed the patient files for the clinical and radiological data that were retrieved at the time of diagnosis before the initial TACE was performed. Clinical and radiological parameters including age, gender, white blood cell count (WBC), platelet (PLT) count, alanine transaminase (ALT), aspartate aminotransferase (AST), total bilirubin (TBIL), indirect bilirubin (IBIL), alkaline phosphatase (ALP), albumin (ALB), C-reactive protein (CRP), AFP, hepatitis B surface antigen (HbsAg), splenomegaly, metastasis, vascular invasion, tumor number, tumor size, antiviral therapy, and tumor-node-metastasis (TNM) stage were collected and analyzed.

### Treatment Procedure

Each patient in this study received three cycles of uniform treatment protocols. The Seldinger technique was performed as previously reported.^30^ Carboplatin at a dose of 300 mg (Bristol-Myers Squibb, NY, New York, USA) was used for hepatic artery infusion chemotherapy. Subsequently, 50 mg epirubicin (Pharmorubicin, Pfizer, Wuxi, Jiangsu, China) and 6 mg mitomycin (Zhejiang Hisun Pharmaceutical Co. Ltd., Taizhou, Zhejiang, China) mixed with Lipiodol (Lipiodol Ultra-Fluide; Andre Guerbet Laboratories, France) were used for chemolipiodolization. The Lipiodol dose was determined based on tumor location, size, and number and ranged from 5 to 30 mL. Sterile-purified H101 viruses were produced for human clinical use by Shanghai Sunway Biotech (Shanghai, China) and safety tested by the National Institute for the Control of Pharmaceutical and Biological Products (Beijing, China). Before the infusion chemotherapy, H101 was injected via catheter into the hepatic artery supplying the tumor(s). A total of 1.0 × 10^12^ virus particles in 10 mL of 0.9% sodium chloride solution were administered.^31^

### Follow-Up

All patients in this study were followed regularly once every 2 months during the first year and once every 3 months thereafter. Radiological examinations, such as liver ultrasonography, computed tomogram (CT) scans, and magnetic resonance imaging (MRI), were performed as needed. Hematological tests, including AFP and liver function, were performed each time. Overall survival (OS) was defined as the duration from the date of the first TACE until death or the last follow-up. The last follow-up date was September 30, 2017. The median follow-up period was 13 months.

### Statistical Analysis

Continuous data are presented as means and ranges and compared using Student’s *t* test. Categorical data are shown as frequencies and proportions and compared using a Chi-square test and Fisher’s exact test. Univariate and multivariate analyses were performed using the Cox regression model and the associated 95% confidence interval (CI) was calculated. OS was analyzed using the KM method. The log-rank test was used to compare the differences between groups. All statistical analyses were performed using SPSS version 22 (SPSS Inc., Chicago, IL, USA). MedCalc software version 11.4.2.0 (http://www.medcalc.be) was used to compare the survival. A two-tailed *P* value < 0.05 was considered statistically significant. The cumulative incidence of overall mortality and cancer-specific mortality was determined by the competing risk analysis. Non-cancer-specific mortality was evaluated as competing mortality in this study. The combined effects of the variables on overall mortality and cancer-specific mortality were evaluated by the Cox proportional hazards analysis of the Fine and Grey model.^32^,^33^ Competing risk analysis was performed using R version 3.4.2 software (The R Foundation for Statistical Computing, Vienna, Austria. http://www.r-project.org).

## Results

### Patient Characteristics

This study included 476 HCC patients who received TACE therapy during the study period. Of all the included patients, 238 who received TACE with H101 were sorted into the TACE with H101 group, and the remaining 238 patients who received TACE alone were sorted into the TACE group. Baseline characteristic comparisons between the two groups are shown in Table 1. The clinical data include 430 males (90.3%) and 46 females (9.7%) with a median age of 55 years (range, 15–94 years). Most patients (94.3%) in this cohort were HBsAg-positive. No patients were infected with the hepatitis C virus (HCV). Most patients had an enlarged tumor size (tumor size > 5 cm) and multiple tumors that were identified by radiography. Of the patients, 5.5 and 4.6% had metastases in the TACE with H101 and TACE groups, respectively. The proportion of HBsAg positivity was slightly higher in the TACE group than in the TACE with H101 group. Other than this, only TBIL significantly differed between the two groups.

**Table 1:** The relationship between clinicolpathological factors and TACE therapy combined with H101 or not

Charateristics		*N*	TACE therapy		*P*
			Without H101	With H101	
Total		476	238	238	
Age	< 60	337	164	173	0.420
	≥ 60	139	74	65	
Gender	Male	430	214	216	0.877
	Female	46	24	22	
WBC (× 10^9^/L)	< 10	446	222	224	0.851
	≥ 10	30	16	14	
PLT (× 10^9^/L)	< 10	58	35	23	0.195
	10~300	367	176	191	
	≥ 300	51	27	24	
ALT (U/L)	< 40	168	78	90	0.291
	≥ 40	308	160	148	
AST (U/L)	< 45	163	79	84	0.699
	≥ 45	313	159	154	
ALP (U/L)	< 100	203	105	98	0.578
	≥ 100	273	133	140	
GGT (U/L)	< 50	83	41	42	1.000
	≥ 50	393	197	196	
ALB (g/L)	< 35	50	25	25	1.000
	≥ 35	426	213	213	
TBIL (mmol/L)	< 20.5	379	180	199	0.040
	≥ 20.5	97	58	39	
CRP (mg/L)	< 8	248	123	125	0.927
	≥ 8	228	115	113	
HBsAg	Negative	27	19	8	0.046
	Positive	449	219	230	
AFP (ng/ml)	< 400	262	122	140	0.117
	≥ 400	214	116	98	
Splenomegaly	Absent	311	148	163	0.177
	Present	165	90	75	
Metastasis	Absent	452	227	225	0.835
	Present	24	11	13	
Vascular invasion	Absent	337	170	167	0.840
	Present	139	68	71	
Tumor number	Single	172	91	81	0.391
	Multiple	304	147	157	
Tumor size (cm)	< 5	127	65	62	1.000
	≥ 5	346	173	173	
Antivirus therapy	No	251	115	136	0.066
	Yes	225	123	102	
TNM stage	I	131	67	64	0.967
	II	68	34	34	
	IIIA	126	65	61	
	IIIB	127	61	66	
	IVB	24	11	13	

*TACE* transarterial chemoembolization, *WBC* white blood cell count, *PLT* platelet, *ALT* alanine transaminase, *AST* aspartate aminotransferase, *ALP* alkaline phosphatase, *GGT* gamma-glutamyl transpeptidase, *ALB* albumin, *TBIL* total bilirubin, *CRP* C-reactive protein, *AFP* alpha-fetoprotein, *TNM* tumor-node-metastasis

### Survival Data

For the entire study cohort, the estimated 1-, 2-, and 3-year OS rates were 58.4, 36.5, and 26.2%, respectively. To the time of the last follow-up, 289 patients died (60.7%), including 273 cancer-specific deaths and 16 non-cancer-specific deaths. Regarding non-cancer-specific deaths, 10/16 patients (62.5%) died from treatment-related comorbidities, 4/16 patients (25%) died from cardiovascular disease, and 2/16 patients (12.5%) died from accidents.

### OS Analysis

**Fig. 1:**
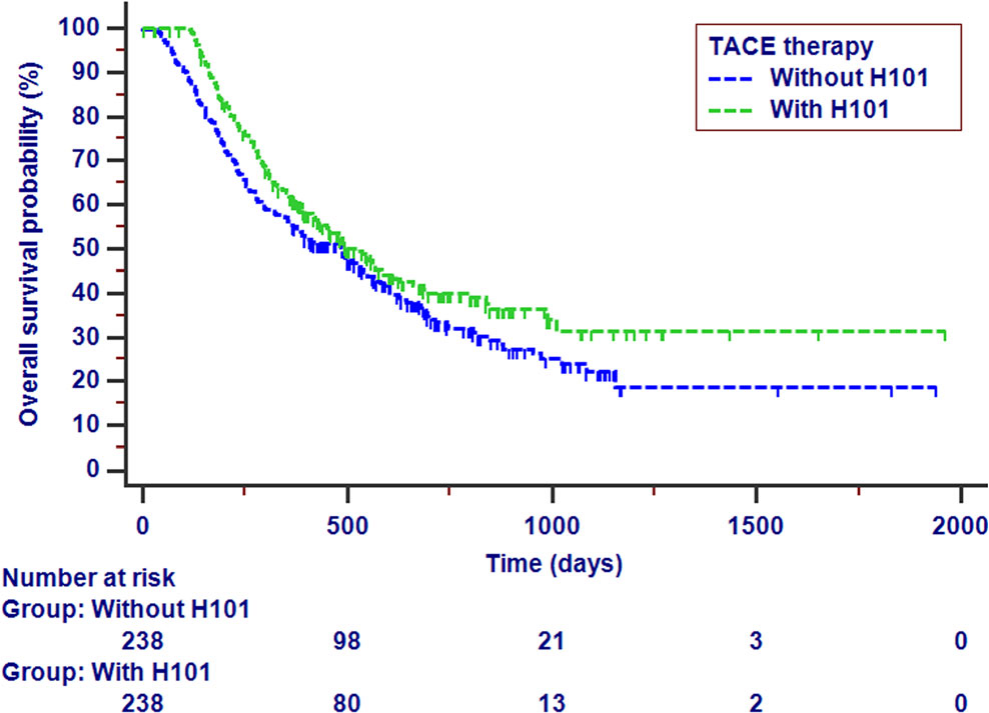
Kaplan-Meier OS curve stratified by TACE therapy combined with H101 or not for patients with HCC (*P* = 0.047). Abbreviations: *OS*, overall survival; *TACE*, transarterial chemoembolization; *HCC*, hepatocellular carcinoma

The patients in TACE with H101 group had significantly favorable prognoses compared with patients in the TACE group (*P* = 0.047, Fig. 1). For patients in the TACE with H101 group, the estimated 1-, 2-, and 3-year OS rates were 61.0, 40.0, and 31.5%, respectively, while for patients in the TACE group, the estimated 1-, 2-, and 3-year OS rates were 55.0, 33.4, and 22.3%, respectively. The median OS for patients in the TACE with H101 and TACE groups were 13.7 and 13.1 months, respectively. Patients with elevated AFP values, metastases, vascular invasion, multiple tumors, larger tumors, and elevated TNM stages had poorer OS based on the univariate analysis. Apart from these variables, age, antiviral therapy, and combined TACE and H101 therapy were all associated with OS (Table 2).

**Table 2:** Univariate analysis for OS in the study cohort

Characteristic		HR (95% CI)	*P*
Age	< 60/≥ 60	0.657(0.501–0.860)	0.002
Gender	Male/Female	1.253(0.863–1.819)	0.236
HBsAg	Negative/Positive	0.663(0.416–1.056)	0.084
AFP	< 400/≥ 400	1.758(1.361–2.272)	< 0.001
Splenomegaly	Absent/Present	1.189(0.934–1.512)	0.159
Metastasis	Absent/Present	3.746(2.428–5.779)	< 0.001
Vascular invasion	Absent/Present	2.159(1.697–2.746)	< 0.001
Tumor number	Single/Multiple	1.298(1.013–1.661)	0.039
Tumor size (cm)	< 5/≥ 5	4.683(3.252–6.743)	< 0.001
Antivirus therapy	No/ Yes	0.715(0.566–0.903)	0.005
TNM stage	I/ II/ IIIA/ IIIB/ IVB	1.560(1.409–1.726)	< 0.001
TACE therapy	Without H101/ With H101	0.791(0.626–0.998)	0.048

*OS* overall survival, *HR* hazard ratio, *CI* confidence interval; other abbreviations as in Table 1

### Competing Risk Analysis

**Fig. 2:**
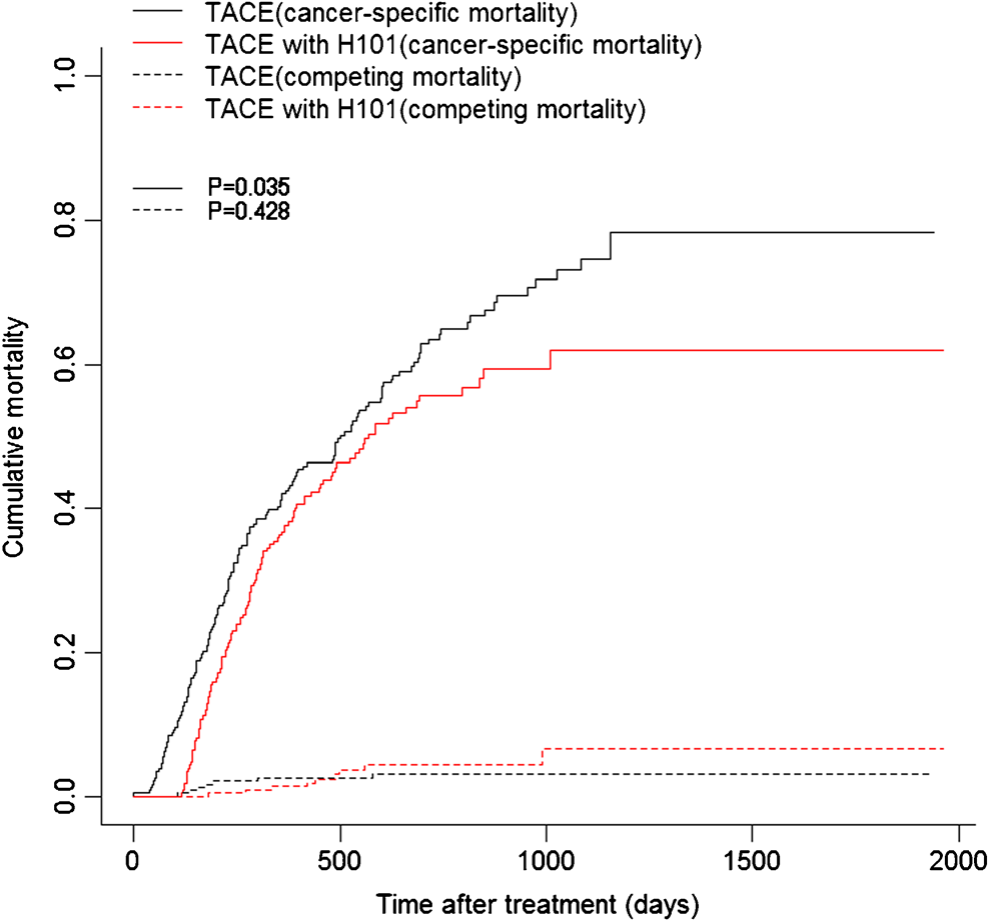
Cumulative cancer-specific and competing mortality curves stratified by TACE therapy combined with H101 or not for patients with HCC. Abbreviations: *TACE*, transarterial chemoembolization; *HCC*, hepatocellular carcinoma

**Fig. 3:**
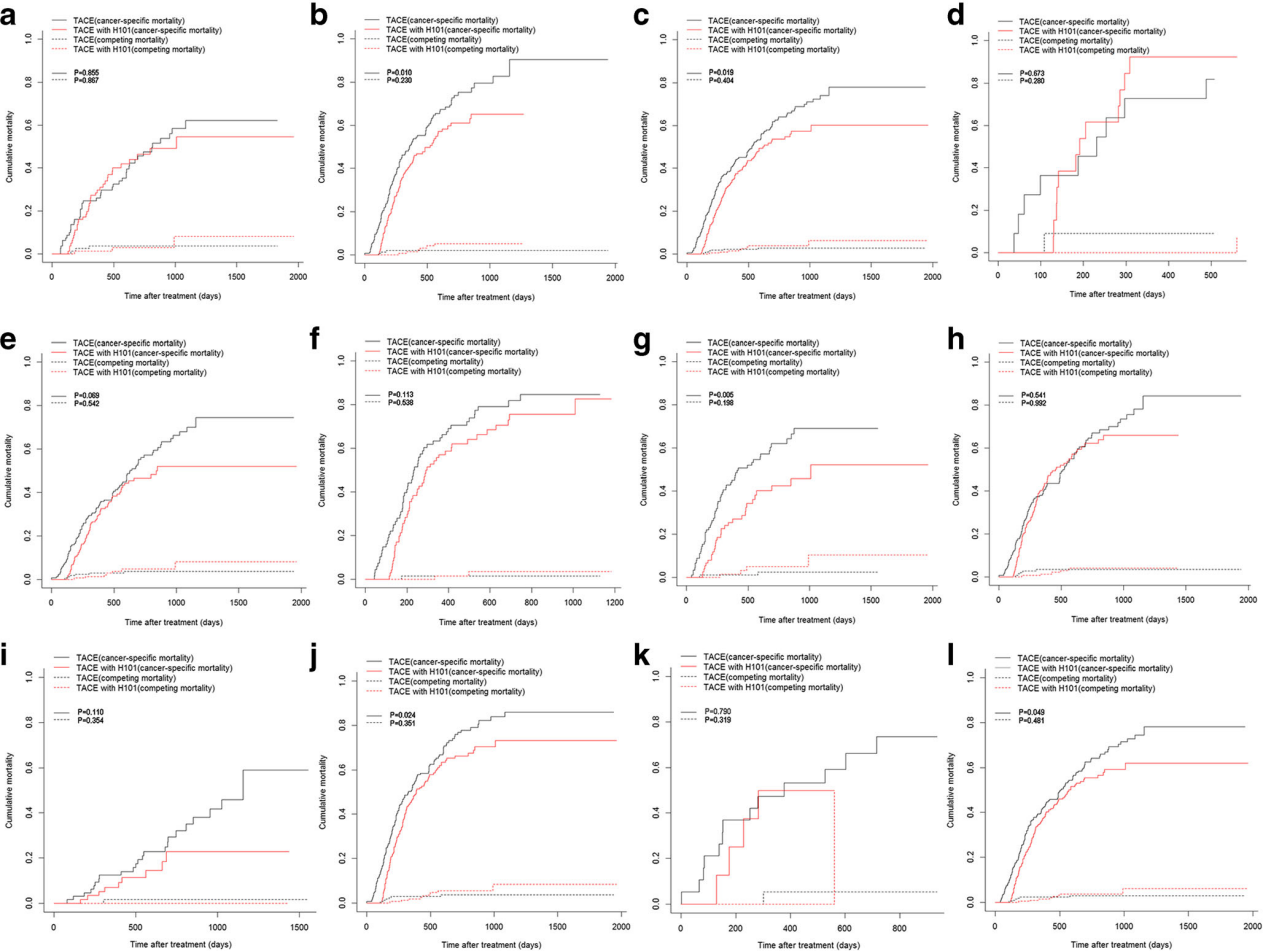
Cumulative cancer-specific and competing mortality curves stratified by TACE therapy combined with H101 or not for patients with HCC in subgroup of non-elevated AFP (**a**), elevated AFP (**b**), absence of metastasis (**c**), presence of metastasis (**d**), absence of vascular invasion (**e**), presence of vascular invasion (**f**), single tumor (**g**), multiple tumors (**h**), tumor size less than 5 cm (**i**), tumor size more than 5 cm (**j**), HBsAg negative (**k**), HBsAg positive (**l**). Abbreviations: *TACE*, transarterial chemoembolization; *AFP*, alpha-fetoprotein; *HCC*, hepatocellular carcinoma; *HBsAg*, hepatitis B surface antigen

For all included patients, the univariate competing risk analysis showed that the 1-, 2-, and 3-year cancer-specific mortality rates for patients in the TACE with H101 group vs. the TACE group were 37.3 vs. 42.0%, 55.7 vs. 63.5%, and 61.9 vs. 74.7%, respectively (*P* = 0.035, Fig. 2). The median cancer-specific mortality for patients in the TACE with H101 and TACE groups was 18.7 and 16.3 months, respectively. The 1-, 2-, and 3-year cumulative mortality curve showed that the competing mortalities were comparable between the TACE with H101 and TACE groups (1.3 vs. 2.5%, 4.3 vs. 3.0%, and 6.6 vs. 3.1%, respectively, *P* = 0.428, Fig. 2). Competing mortality rates were compared in patient subgroups stratified by AFP, metastasis, vascular invasion, multiple tumor, tumor size, and HBsAg-positive values. In the subgroup competing mortality analyses, the cumulative mortality rates were significantly higher in the TACE group than in the TACE with H101 group when patients had elevated AFP values (*P* = 0.010, Fig. 3b), absence of metastases (*P* = 0.019, Fig. 3c), absence of multiple tumors (*P* = 0.005, Fig. 3g), enlarged tumors (*P* = 0.024, Fig. 3j), or HBsAg positivity (*P* = 0.049, Fig. 3l). Furthermore, no significant differences were found in competing mortality between the two groups in the subgroup analyses (*P* > 0.05).

### Multivariate Analysis

Variables that were significantly associated with OS were analyzed by multivariate Cox regression analysis. Metastasis, vascular invasion, tumor number, and tumor size are all components of the TNM stage system. To avoid multicollinearity, TNM stage was not included in the multivariate analysis. After a stepwise removal of variables, only AFP (hazard ratio (HR), 1.554; 95% CI, 1.193–2.025; *P* = 0.001), metastasis (HR, 2.162; 95% CI, 1.377–3.392; *P* = 0.001), vascular invasion (HR, 1.532; 95% CI, 1.191–1.969; *P* = 0.001), tumor size (HR, 4.029; 95% CI, 2.773–5.854; *P* < 0.001), antiviral therapy (HR, 0.783; 95% CI, 0.616–0.995; *P* = 0.045), and TACE therapy combined with H101 or not (HR, 0.688; 95% CI, 0.544–0.870; *P* = 0.002) significantly predicted OS (Table 3). In addition, the multivariate competing risks (Fine and Gray approach) and Cox analyses were conducted for all patients. Adjusted HRs are shown in Table 3. AFP (HR, 1.546; 95% CI, 1.175–2.030; *P* = 0.002), vascular invasion (HR, 1.655; 95% CI, 1.251–2.190; *P* < 0.001), tumor size (HR, 3.593; 95% CI, 2.540–5.080; *P* < 0.001), and TACE therapy combined with H101 or not (HR, 0.668; 95% CI, 0.518–0.860; *P* = 0.002) were independent factors in decreasing cancer-specific mortality.

**Table 3:** Multivariate analysis for OS and cancer-specific mortality in the study cohort

Characteristic		OS		Cancer-specific mortality	
		HR (95% CI)	*P*	HR (95% CI)	*P*
Age	< 60/≥ 60	0.860(0.653–1.133)	0.284	0.840(0.626–1.130)	0.240
AFP	< 400/≥ 400	1.554(1.193–2.025)	0.001	1.546(1.175–2.030)	0.002
Metastasis	Absent/Present	2.162(1.377–3.392)	0.001	1.779(0.959–3.300)	0.068
Vascular invasion	Absent/Present	1.532(1.191–1.969)	0.001	1.655(1.251–2.190)	< 0.001
Tumor number	Single/Multiple	1.210(0.938–1.560)	0.143	1.211(0.925–1.580)	0.160
Tumor size (cm)	< 5/≥ 5	4.029(2.773–5.854)	< 0.001	3.593(2.540–5.080)	< 0.001
Antivirus therapy	No/Yes	0.783(0.616–0.995)	0.045	0.776(0.601–1.000)	0.053
TACE therapy	Without H101/With H101	0.688(0.544–0.870)	0.002	0.668(0.518–0.860)	0.002

Abbreviations as in Table 2

## Discussion

To our knowledge, this study is the first to demonstrate the prognostic value of H101 combined with TACE in HCC patients using a competing risk analysis. Compared with TACE therapy, patients diagnosed with unresectable HCC and treated with a combination of TACE and H101 therapy had increased OS and decreased cancer-specific mortality in our study. This benefit mainly originated from decreased cancer-specific mortality, consistent with previous reports.^31^,^34^ Furthermore, subgroup analyses were adopted, and comparing cumulative mortality showed that cumulative mortalities differed significantly between the TACE with H101 and TACE groups. In addition, the survival benefit for H101 was more obvious when HCC patients had elevated AFP, no metastases, single tumors, enlarged tumors, or HBsAg positivity.

Survival analysis is often used to assess the time to an event of interest in follow-up studies. Apart from the event of interest, other events may prevent target outcome from occurring. In the HCC patient survival analysis, non-cancer-specific death prevented the appearance of cancer-specific death. In standard survival analysis, the risk of cancer-specific death is incorrect if non-cancer-specific deaths occur.^35^ Therefore, the KM method may overestimate the cumulative mortality of HCC patients who received TACE with or without H101. In this study, non-cancer-specific mortality was accounted for as competing mortality, and cancer-specific mortality was compared with the results from the standard survival analysis.

Competing risk methods are used to analyze risk factors in biomedical research, especially in cancer, either at the screening or treatment stage, which may influence decision making.^36^,^37^ What is more, multivariate Cox regression may lead to confounding in exploring predicted factor values when competing risks are present.^38^ Evaluating factor efficacy will be more realistic using competing risk analysis. Multivariate analysis showed significant differences in both cancer-specific mortalities and overall survival rates between the two groups in this study. The results of the current work may further consolidate the role of H101 in TACE therapy for HCC patients.

H101 is an E1B/E3B-deleted adenovirus that restricts p53-mutated neoplasm replication, sparing p53 wild-type tissues.^39^ The decreased cancer-specific mortality for HCC patients treated with combined TACE and H101 therapy compared with TACE therapy alone may be explained by the following mechanism. Carboplatin, a chemotherapy drug commonly used for TACE at our institution, induces apoptosis and cell cycle arrest through p53 apoptosis.^40^ Tumor cell inhibition will be enhanced when TACE and H101 are used together. In addition, over 80% of patients with HCC in Asia are hepatitis B virus (HBV)-positive^41^ and over 90% of patients in this study are HBV-positive. HBV produces HBV X protein (HBx), which inhibits p53 gene expression.^42^ Therefore, H101 provides a survival benefit for HCC patients. In this study, it was revealed that patients had decreased cancer-specific mortality in TACE with H101 group compared with TACE group when patients were HBV-positive, while the differences were not significant between the two groups when patients were HBV-negative. In addition, some reports have shown that antivirus therapy for HBV-positive patients was associated with prolonged OS after TACE.^43^,^44^ Similarly, antivirus therapy was an independent prognostic factor in both multivariate analyses and the Fine and Gray regression model for HCC patients in this study. It was also suggested that the combining H101 with antivirus therapy may increase curative effects for HCC patients after TACE therapy, which requires further investigation.

Similar to other report,^45^ our study revealed that elevated AFP values, presence of vascular invasion, and enlarged tumors were also independent predictors for HCC patients in this study. Interestingly, subgroup analyses showed that patients who received combined TACE and H101 therapy had significantly decreased cancer-specific mortality in the elevated-AFP and enlarged tumor subgroups. Cancer-specific mortalities in patients after TACE therapy were higher than those of the combined TACE and H101 therapy in the presence of vascular invasion subgroup, although the differences were not significant in this study. Cancer-specific mortalities were comparable between the two groups in patients whose AFP values were lower than 400 mg/mL or whose tumor sizes were smaller than 5 cm in this study. One possible explanation is that the p53 gene mutations and loss of p53 gene heterozygosity are common in HCC, especially HCC with a heavy tumor burden, which is reflected by the elevated APF values and enlarged tumors.^46^,^47^ H101 may be more effective for tumors in which p53 gene mutations or deletions are more frequent.^48^ What is more, blood flow is abundant in HCC with a heavy tumor burden.^49^ Higher H101 concentrations in vascular-rich areas where tumor cells grow faster may inhibit tumor cell growth in a timely and effective manner.

The major limitations of the present study were its retrospective nature and the single-center experiment. In addition, most included patients were predominantly HBV-infected in China. Whether this result can be applied to patients with HCV infection requires further confirmation. Additionally, longer follow-up times may be needed to observe more endpoints to more precisely estimate cancer-specific mortality. Large-scale, further prospective, randomized-controlled, long-term studies are needed to confirm our results.

In conclusion, based on the competing risk model, we demonstrated that combining TACE and H101 therapy decreased cancer-specific mortality in HCC patients compared with TACE therapy alone. The survival benefit was more obvious in patients with elevated AFP, absence of metastasis, single tumor, enlarged tumor, and HBsAg positivity. The results of this study may further consolidate the role of H101 in TACE therapy for patients with HCC.
